# Sodium salicylate ameliorates exercise-induced muscle damage in mice by inhibiting NF-kB signaling

**DOI:** 10.1186/s13018-023-04433-w

**Published:** 2023-12-15

**Authors:** Yiming Wang, Yuning Sun, Chunhui Yang, Bing Han, Sining Wang

**Affiliations:** 1 Department of Sports, Northeastern University, Lane 3, Wenhua Road, Heping District, Shenyang City, 110819 China; 2 Department of General, Huanggu District People’s Government Office, Shenyang City, 110032 China

**Keywords:** Eccentric exercise, Inflammation, Muscle damage, Sodium salicylate

## Abstract

**Background:**

Eccentric muscle contraction can cause muscle damage, which reduces the efficiency of exercise. Previous evidence suggested that Sodium salicylate (SS) could improve the repair of aged muscle. This study intends to investigate whether SS can impact skeletal muscle damage caused by eccentric exercise.

**Methods:**

Eccentric treadmill exercise was performed to induce muscle damage in mice. Plasma levels of muscle damage markers were estimated. RT-qPCR was employed for detecting mRNA levels of proinflammatory mediators in murine gastrocnemius muscle. Immunofluorescence staining of laminin/DAPI was utilized for quantifying centrally nucleated myofibers in the gastrocnemius muscle. Western blotting was implemented to examine protein levels of mitsugumin 53 (MG53), matrix metalloproteinase (MMP)-2/9, and NF-κB signaling-related markers.

**Results:**

SS administration reduced muscle damage marker production in the plasma and decreased the levels of proinflammatory mediators, MG53 and MMP-2/9 in mice after exercise. SS alleviated the severity of muscle damage in the gastrocnemius of mice after eccentric exercise. SS blocked NF-κB signaling pathway in the gastrocnemius muscle.

**Conclusion:**

SS administration ameliorates skeletal muscle damage caused by eccentric exercise in the mouse model.

**Supplementary Information:**

The online version contains supplementary material available at 10.1186/s13018-023-04433-w.

## Introduction

Eccentric muscle contractions occur when the force applied to the muscle exceeds the force generated instantly by the muscle itself, which are featured with the lengthening of skeletal muscle [1]. Mounting evidence has demonstrated that eccentric exercise, especially when unaccustomed, induces muscle damage due to intense muscle contraction [2]. Muscle damage results in inflammatory cell infiltration into the damaged site, cell membrane destruction, and extracellular matrix degradation [3]. Excessive inflammatory response is responsible for secondary tissue damage [4]. Evidence suggests that elevated ECM degradation impedes force transmission following muscle damage [5]. Matrix metalloproteinases (MMPs) are a family of zinc-dependent proteolytic enzymes that can degrade various ECM components [6].

Sodium salicylate (SS) is a nonsteroidal anti-inflammatory drug that has been indicated to play pivotal roles in various pathological processes [7]. Choi et al. demonstrated that SS facilitates the browning of white adipocytes by enhancing HO-1 to promote M2 macrophage polarization [8]. A low dose of SS contributes to ovulation in animal models by upregulating CYP17A1 [9]. Importantly, a previous report indicated that SS could improve the repair of aged muscle [10]. Nonetheless, it is unanswered whether SS has an impact on muscle damage induced by eccentric exercise.

Nuclear factor kappa B (NF-κB) is a transcription factor known to regulate immune response and inflammation [11]. In response to stimuli, the inhibitor of NF-κB kinase subunit beta (IKKβ) is activated and phosphorylates NF-κB inhibitor alpha (IκBα), which triggers its degradation and subsequently leads to NF-κB activation [12]. Activated NF-κB translocates into the nucleus and promotes inflammatory cytokine transcription [13]. Previous evidence has suggested that an acute bout of exercise increases NF-κB signaling in animals [14]. Intriguingly, SS is widely recognized to be an inhibitor of NF-κB signaling [15].

This study intended to investigate the functions of SS on eccentric exercise-triggered muscle damage in a mouse model. It was hypothesized that SS might alleviate the inflammatory response in mice after eccentric exercise by mediating NF-κB signaling. Our results might help to develop new methods for improving muscle damage caused by intense exercise.

## Materials and methods

### Mice

Male C57BL/6 mice (6–7 weeks, 18–23 g; Cavens, Changzhou, China) were housed in a temperature- and humidity-controlled environment (22 ± 1 ℃, 40–70%) with 12-h light/dark cycles and free access to food and water. The mice were allowed for one-week acclimatation before experiments. All animal experiments were performed as per the NIH Guide for the Care and Use of Laboratory Animals. Approval of the study was obtained from the Ethical Committee of Northeastern University.

### Experimental design

Thirty-two mice were randomly assigned to 4 groups (8 mice/group): control, SS; exercise (EX) and EX+SS. All mice were trained to run on a treadmill at a speed of 10 m/min, 10 min/day, three times a week. One hour before exhaustive exercise, mice in the SS and EX+SS groups were administrated with SS (120 mg/kg, MedChemExpress, Shanghai, China) via oral gavage, while mice in the other two groups received the same amount of normal saline. The dose of SS was determined based on previous reports [16]. Afterward, mice in EX and EX+SS groups performed eccentric exercise (downhill running) on the treadmill (− 16°, 16 m/min) for 60 min. The eccentric exercise conditions were selected based on previous reports [17, 18].

### Sample collection

Twelve hours after exercise, mice (*n* = 4 from each group) were fasted for 12 h, and then blood samples were collected from the abdominal artery. After centrifugation at 1600 × g for 10 min, the resulting plasma samples were stored at – 80 ℃ for subsequent analysis. After blood collection, the mice were sacrificed by cervical dislocation under anesthesia. Gastrocnemius muscles were excised from each group, and immediately snap-frozen in liquid nitrogen and stored at – 80 ℃. The remaining 4 mice in each group were sacrificed 48 h after exercise. Gastrocnemius muscles were collected, embedded in optimal cutting temperature compound, and frozen in liquid nitrogen-cooled isopentane for immunofluorescence staining.

### Plasma biochemical evaluation

The plasma levels of glucose, creatine kinase (CK), lactate dehydrogenase (LDH), alanine aminotransferase (ALT), aspartate aminotransferase (AST), urea nitrogen (BUN) and non-esterified fatty acid (NEFA) were determined using an automatic biochemical analyzer (Roche Modular P800, Roche Diagnostics, Indianapolis, IN).

### Immunofluorescence staining

The frozen muscle samples were sectioned into 10-μm-thick slices using a cryostat microtome (Leica Biosystems, Shanghai, China). The cryosections were then fixed in 4% paraformaldehyde for 15 min, washed twice with PBS, and incubated with blocking buffer (Beyotime, Shanghai, China) for 1 h. Next, the sections were incubated with anti-laminin primary antibody (ab7463, Abcam, Shanghai, China) at 4℃ overnight and washed three times before incubation with Goat Anti-Rabbit IgG H&L (Alexa Fluor® 647) secondary antibody (ab150083, Abcam) at room temperature for 1 h. The sections were mounted with antifade mounting medium containing DAPI (Vector Laboratories, Newark, CA) for nuclear labeling. Images were captured with a fluorescence microscope (Olympus, Tokyo, Japan). Consecutive fields from whole muscle cross-sections were obtained with red channel for laminin and blue channel for DAPI. The centrally nucleated myofibers in the whole muscle cross-section were quantified by counting the number of DAPI-positive stains inside muscle fibers using CellProfiler software (version 4.2.5).

### Reverse transcription quantitative polymerase chain reaction (RT-qPCR)

Total RNA isolation from gastrocnemius muscles was conducted using TRIzol reagent (Invitrogen, Carlsbad, CA). cDNA was prepared by reverse transcription of RNA using iScript cDNA Synthesis Kit (Bio-Rad, Hercules, CA). Real-time qPCR was carried out using SYBR Green PCR Mastermix (Solarbio, Beijing, China) on an ABI 7900 system (Applied Biosystems, Waltham, MA). Normalized to GAPDH, relative mRNA expression was evaluated using the 2^−ΔΔCt^ method. Primer sequences are listed in Additional file 1: Table S1.

### Western blotting

Proteins were extracted from gastrocnemius muscles using RIPA buffer (Solarbio) and quantified with a bicinchoninic acid assay kit (Beyotime). Equal amounts of proteins from each group were resolved in 10% SDS-PAGE, blotted onto polyvinylidene fluoride membranes (Beyotime) and blocked with 5% defatted milk in Tris-buffered saline and Tween-20 (TBST). Then, the membranes were incubated with primary antibodies (shown in Additional file 1: Table S2; Abcam) at 4 ℃ overnight and washed thrice with TBST before incubating with the HRP-conjugated secondary antibody (ab205718, 1:2000, Abcam) for 2 h at room temperature. Lastly, blot signaling was visualized using an ECL detection kit (Solarbio) and evaluated using ImageJ software.

### Statistical analysis

Differences among different treatment groups were analyzed by one-way ANOVA with Tukey’s post hoc analysis using GraphPad Prism 8.0.2 software (GraphPad, San Jose, CA). Data were expressed as the mean ± standard deviation. *p* < 0.05 depicted statistical significance.

## Results

### SS reduces plasma levels of muscle damage markers in mice after exercise

To assess the extent of muscle damage, muscle damage markers in the plasma were detected 24 h after eccentric exercise. As shown in Table 1, plasma glucose level was significantly decreased in both EX and EX+SS groups due to the energy expenditure, along with the increased levels of BUN and NEFA. Moreover, the levels of muscle damage markers, including LDH, CK, AST and ALT, were prominently elevated in the EX group, while SS treatment reduced the levels of these markers, indicating that SS could attenuate eccentric exercise-triggered muscle damage in mice.

**Table 1:** Plasma biochemical analysis

marker	Control	SS	EX	EX+SS
Glucose (mg/dL)	232.5 ± 12.7a	220.3 ± 11.8a	175.3 ± 21.6b	197.4 ± 19.5c
NEFA (µmol/L)	2.1 ± 0.4a	2.2 ± 0.3a	3.2 ± 0.1b	2.6 ± 0.3c
BUN (mg/dL)	22.5 ± 1.7a	23.5 ± 1.7a	34.8 ± 2.2b	28.4 ± 2.4c
CK (IU/L)	948.3 ± 262.1a	812.6 ± 252.1a	5362.7 ± 825.7d	4122.6 ± 925.3c
LDH (IU/L)	442.8 ± 54.7a	409.2 ± 54.7a	1688.3 ± 168.3d	1421.3 ± 215.2c
ALT (IU/L)	32.5 ± 2.1a	32.8 ± 4.3a	125.2 ± 16.8d	87.6 ± 18.6c
AST (IU/L)	119.6 ± 15.6a	155.4 ± 25.7a	503.4 ± 52.8d	392.5 ± 50.7c

*n* = 4 per group. Means with different superscripts (a, b, c, d) are significantly different. ^b^*p* < 0.01, ^d^*p* < 0.001 vs. control group, ^c^*p* < 0.05 vs. EX group

### SS alleviates eccentric exercise-triggered inflammation in mice

**Fig. 1:**
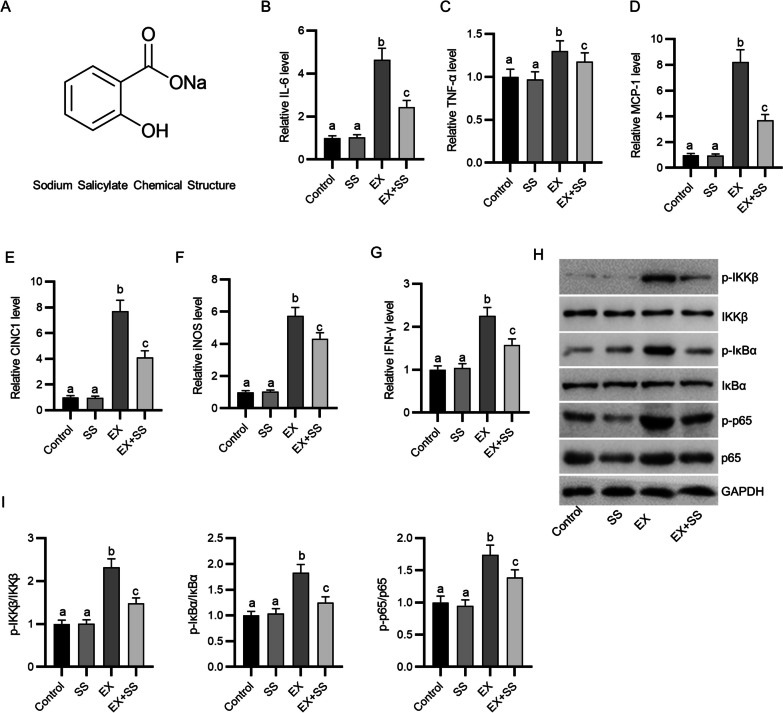
SS alleviates exhaustive exercise-triggered inflammation in mice. **A** Chemical structure of SS. **B**–**G** RT-qPCR for detecting mRNA levels of proinflammatory mediators in the gastrocnemius muscle of each group (*n* = 4). **H–I** Western blotting for measuring NF-κB signaling-related protein levels in the gastrocnemius muscle of each group (*n* = 4). Means with different superscripts (a, b, c) are significantly different. ^b^*p* < 0.01 vs. control group; ^c^*p* < 0.01 vs. EX group

To examine the impact of SS on inflammatory response in skeletal muscle, we measured the mRNA levels of proinflammatory mediators in the gastrocnemius muscle, including IL-6, TNF-α, MCP-1, CINC-1, IFN-γ and iNOS. As depicted by the results, SS administration markedly abated intense exercise-induced upregulation of these proinflammatory mediators (Fig. 1B–G). Moreover, for exploration of the potential mechanism underlying SS-mediated protection against inflammation in the gastrocnemius muscle, we estimated its effect on the prototypical proinflammatory NF-κB signaling pathway. Consistent with the above results, western blotting displayed that intense exercise markedly promoted phosphorylation of IκBα, IKKβ and p65 in murine gastrocnemius muscle, indicating that intense exercise triggered activation of NF-κB signaling. However, the effects evoked by intense exercise were shown to be reversed by SS administration (Fig. 1H–I). Collectively, the above data revealed that SS administration attenuates intense treadmill exercise-triggered inflammatory response in the gastrocnemius muscle of mice.

### SS alleviates the severity of muscle injury in the gastrocnemius

**Fig. 2:**
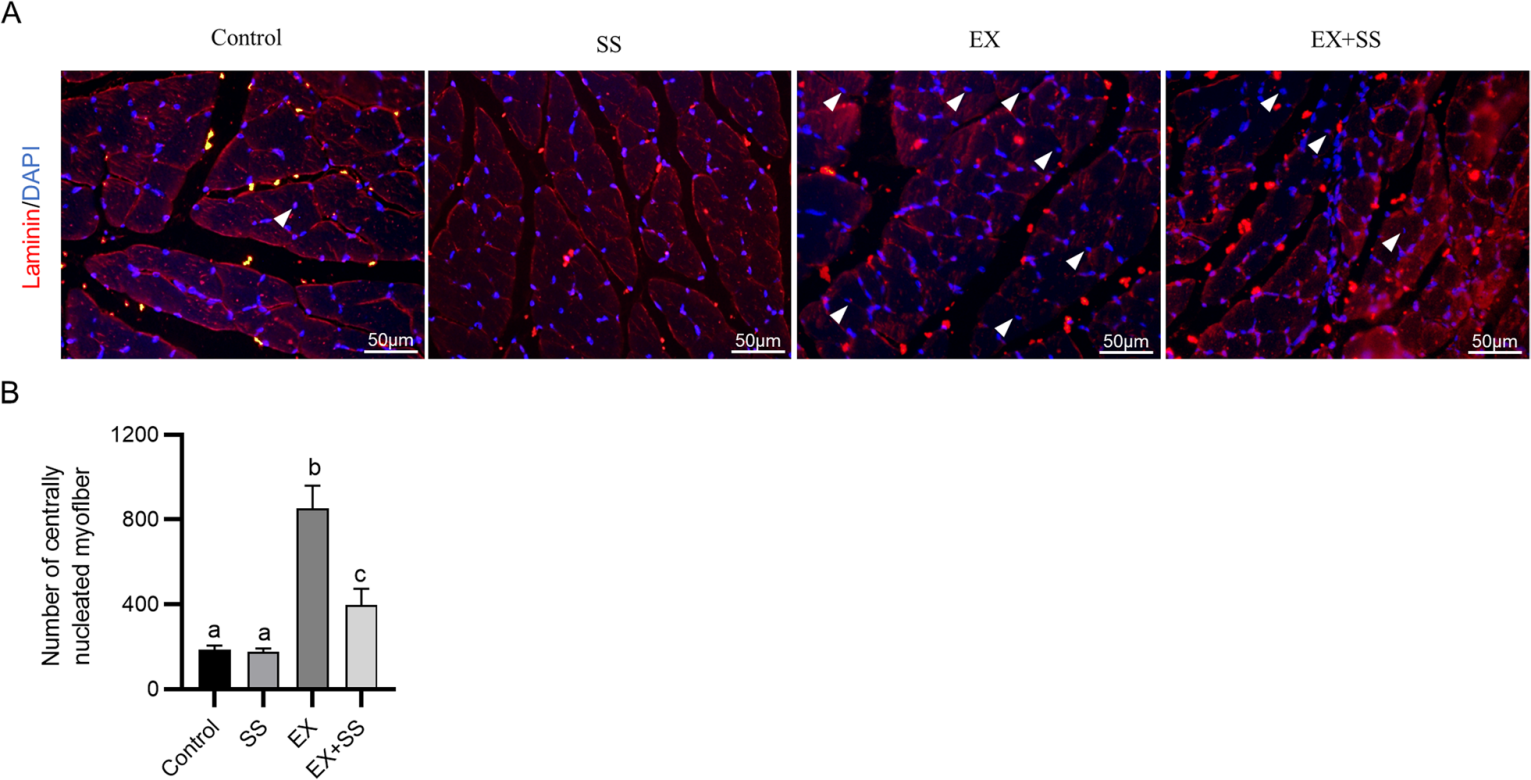
SS alleviates the severity of muscle injury in the gastrocnemius. **A** Representative immunofluorescence images of the gastrocnemius muscle stained with laminin (red) and DAPI (blue) in each group (48 h after exercise). **B** Quantification of centrally nucleated fibers (*n* = 4). Means with different superscripts (a, b, c) are significantly different. ^b^*p* < 0.001 vs. control group; ^c^*p* < 0.001 vs. EX group

Damaged skeletal muscle has the ability to regenerate, and muscle regeneration is manifested by the presence of central nuclei. To evaluate the severity of exercise-induced muscle damage, immunofluorescence staining was carried out to detect centrally nucleated myofibers in the gastrocnemius muscle from each group. As shown by the results, in unexercised mice, there were only a very small number of centrally nucleated fibers in the gastrocnemius muscle. After eccentric exercise, the number of central nuclei in the gastrocnemius muscle was significantly increased (Fig. 2A–B). However, the number of centrally nucleated myofibers was prominently decreased in the EX+SS group in comparison to that in the EX group (Fig. 2A–B), indicating that SS administration alleviated the severity of skeletal muscle damage.

### SS alleviates exercise-triggered upregulation of MG53 and MMPs in the gastrocnemius muscle

**Fig. 3:**
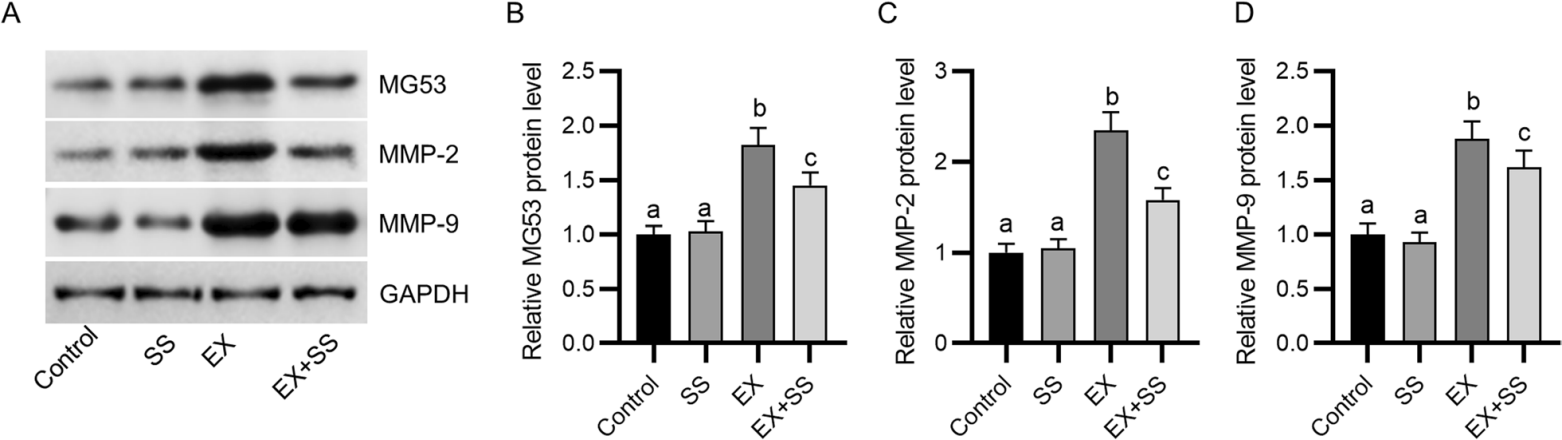
SS alleviates exercise-triggered muscle structure destruction. **A**–**D** Western blotting for evaluating protein levels of MG53, MMP-2, and -9 in the gastrocnemius muscle of each group (*n* = 4). Means with different superscripts (a, b, c) are significantly different. ^b^*p* < 0.01 vs. control group; ^c^*p* < 0.01 vs. EX group

Western botting was carried out to assess the levels of structural proteins and MMPs in gastrocnemius muscle after exercise. As displayed by the results, eccentric exercise significantly elevated the levels of mitsugumin 53 (MG53), MMP-2 and MMP-9, whereas these effects were partially counteracted by SS administration (Fig. 3A–D). These indicated that administration of SS might alleviate exercise-evoked muscle membrane destruction and ECM degradation.

## Discussion

Eccentric exercise provides a potent stimulus to enhance muscle strength, however, it can easily result in muscle damage, which weakens the efficiency of muscle contraction [19]. Exercise-triggered muscle damage is tightly linked to muscle soreness increase, muscle strength decrease, and elevation of muscle-specific circulatory proteins [20]. Many efforts have been made to maintain the benefits of eccentric exercise while attenuating muscle damage. Here, we selected a -16° incline for 60 min of eccentric exercise, which was previously reported to induce a significant decrease in muscle contractile force in mice [17, 18]. The present study revealed that administration of SS could reduce plasma levels of muscle damage markers, alleviate excessive inflammatory response, and ameliorate muscle damage in mice undergoing eccentric exercise.

Damage to muscle fiber membranes leads to the release of some enzymes or proteins such as CK, LDH, and BUN, which indicate the status of muscle injury [21]. Consistent with previous reports [17], our study depicted that eccentric exercise elevated plasma levels of muscle damage markers, while SS treatment markedly restored the effects in mice after exercise. Additionally, our results demonstrated that SS administration alleviated the severity of muscle damage in the gastrocnemius of mice after exercise, as evidenced by the decreased number of centrally nucleated myofibers. A previous study indicated that SS contributed to the repair of aged muscle [10], which partially supported our findings.

After muscle damage, the production and release of inflammatory mediators, such as IL-6, MCP-1, and iNOS were increased, which contributes to secondary muscle injury [22]. The induction of iNOS in skeletal muscle is a hallmark of muscle damage induced by eccentric exercise [23]. SS is well-known to be an anti-inflammatory drug [24]. Consistently, our results depicted that SS treatment markedly abated exercise-induced upregulation of proinflammatory cytokines and chemokines. Furthermore, many studies have suggested that SS exerts its anti-inflammatory activity by repressing the NF-κB signaling pathway [15, 25]. NF-κB signaling is required to modulate the transcription of proinflammatory cytokines in skeletal muscle in response to exercise stress [14]. Similar to previous reports, our results depicted that SS prominently reversed exercise-triggered activation of NF-κB signaling in murine gastrocnemius muscle. In addition, previous evidence has suggested that the role of anti-inflammatory drugs in muscle repair is complicated by differences in time point(s) chosen for evaluation [26]. Therefore, further investigations are needed to evaluate the effect of SS on muscle damage at various time points, which helps to elucidate its role in muscle damage recovery.

MG53, also known as tripartite motif containing 72 (TRIM72), is highly expressed in striated muscle and has been indicated to exert a protective effect on multiple organs after ischemia/reperfusion injury [27]. MG53 is released as a myokine after exercise and is essential for sarcolemmal membrane repair [28]. Here, we found that MG53 protein was prominently decreased in SS-administrated mice after exercise, indicating that muscle damage was less severe in the SS-treated group than the group without SS treatment. Moreover, previous evidence has illuminated that muscle damage results in the degradation of ECM [29]. Overproduction of MMPs aggravates tissue degeneration, consequently impairing myogenesis [30]. Enhanced levels of MMP-2 and -9 have been observed after eccentric muscle contraction [31]. Similar results were depicted in our study. In addition, SS administration reduced MMP-2 and -9 expression in the gastrocnemius muscle of mice after exercise, indicating that SS might protect against eccentric muscle contraction-evoked ECM degradation. A previous report indicated that SS could repress MMP-9 expression in tumor cells without affecting MMP-2 expression [32].

In conclusion, this study reveals that SS administration improves eccentric exercise-triggered muscle damage in mice. Additionally, SS alleviates inflammatory response in mice after exercise probably by repressing NF-κB signaling pathway. Our findings might help develop new methods for improving muscle damage caused by eccentric muscle contraction.

### Supplementary Information

**Additional file 1.** Primer sequences (Table S1) and primary antibodies (Table S2) used in this study.

## Data Availability

The datasets used or analyzed during the current study are available from the corresponding author on reasonable request.
