# Supplementary material for: Sodium salicylate ameliorates exercise-induced muscle damage in mice by inhibiting NF-kB signaling
Source: J Orthop Surg Res. 2023 Dec 15;18:967. doi: 10.1186/s13018-023-04433-w (PMC10722820; doi:10.1186/s13018-023-04433-w)
Supplement: Supplementary file 1 — Additional file 1. Primer sequences (Table S1) and primary antibodies (Table S2) used in this study. [file 13018_2023_4433_MOESM1_ESM.docx]

**Table S1. Primers used in RT-qPCR.**

| Gene | Forward (5’-3’) | Reverse (5’-3’) |
| --- | --- | --- |
| IL-6 | GATTCAATGAGGAGACTTGCC | TGTTCTGGAGGTACTCTAGGT |
| TNF-α | CTCTAATCAGCCCTCTGGC | GAGGGTTTGCTACAACATGG |
| MCP-1 | CCAGATGCAATCAATGCCC | TGGTCTTGAAGATCACAGCT |
| CINC-1 | CCCAAGAACATCCAAAGTGTG | CATTCTTGAGTGTGGCTATGAC |
| iNOS | ATGACCTTCAGTATCACAACCT | CTGGAGACTTCTTTCCCGT |
| IFN-γ | CATCAAGGAAGACATGAATGTC | GACATTCAAGTCAGTTACCGA |

**Table S2. Primary antibodies used in western blotting.**

| Target | Host species/clonality | Cat. No | Concentration |
| --- | --- | --- | --- |
| IκBα | Rabbit monoclonal | ab32518 | 1:1000 |
| p-IκBα | Rabbit monoclonal | ab133462 | 1:10000 |
| IKKβ | Rabbit monoclonal | ab124957 | 1:1000 |
| p-IKKβ | Rabbit polyclonal | ab194528 | 1:500 |
| p65 | Rabbit monoclonal | ab32536 | 1:1000 |
| p-p65 | Rabbit monoclonal | ab76302 | 1:1000 |
| MG53 | Rabbit monoclonal | ab307593 | 1:1000 |
| MMP-2 | Rabbit monoclonal | ab92536 | 1:1000 |
| MMP-9 | Rabbit monoclonal | ab228402 | 1:1000 |
| GAPDH | Rabbit polyclonal | ab9485 | 1:2500 |
